# Pretreatment and gaseous radiocarbon dating of 40–100 mg archaeological bone

**DOI:** 10.1038/s41598-019-41557-8

**Published:** 2019-03-29

**Authors:** H. Fewlass, T. Tuna, Y. Fagault, J.-J. Hublin, B. Kromer, E. Bard, S. Talamo

**Affiliations:** 1 Department of Human Evolution, Max Planck Institute for Evolutionary Anthropology, Deutscher platz 6, D-04103 Leipzig, Germany; 2 CEREGE, Aix Marseille Univ, CNRS, IRD, INRA, Collège de France, Technopôle de l’Arbois, BP 80, 13545 Aix-en-Provence, France; 3 Institute of Environmental Physics, University of Heidelberg, INF 229, D-69120 Heidelberg, Germany

## Abstract

Radiocarbon dating archaeological bone typically requires 300–1000 mg material using standard protocols. We report the results of reducing sample size at both the pretreatment and ^14^C measurement stages for eight archaeological bones spanning the radiocarbon timescale at different levels of preservation. We adapted our standard collagen extraction protocol specifically for <100 mg bone material. Collagen was extracted at least twice (from 37–100 mg material) from each bone. Collagen aliquots containing <100 μg carbon were measured in replicate using the gas ion source of the AixMICADAS. The effect of sample size reduction in the EA-GIS-AMS system was explored by measuring ^14^C of collagen containing either *ca*. 30 μg carbon or *ca*. 90 μg carbon. The gas dates were compared to standard-sized graphite dates extracted from large amounts (500–700 mg) of bone material pretreated with our standard protocol. The results reported here demonstrate that we are able to reproduce accurate radiocarbon dates from <100 mg archaeological bone material back to 40,000 BP.

## Introduction

Bone is one of the most frequently radiocarbon-dated materials recovered from archaeological sites. However, many precious archaeological bones, such as human remains or Palaeolithic bone tools, are too small or valuable for extensive destructive sampling. The reduction of sample size to enable direct dating of precious bone is therefore a key concern for the archaeological community.

In the 1960s and 1970s, gas proportional counters required many grams of bone to produce a radiocarbon date^1,2^. The development and utilisation of Accelerator Mass Spectrometers (AMS) in the 1980s represented a revolutionary step in the reduction of sample size and time required for dating^3^. Routine measurements today typically require 500–1000 micrograms of carbon (μg C) to produce a high precision date. In recent years, several AMS labs have worked on modifications to the graphitisation and AMS measurement process for smaller samples containing <500 μg C^4–13^. However, the graphitisation of small sample sizes is often time consuming and can be prone to large contamination effects^14,15^. A recent study by Cersoy, *et al*.^16^ demonstrated that graphite targets containing *ca*. 200 μg C from archaeological bones can be successfully produced and measured using the IonPlus Automated Graphitisation Equipment III (AGE 3)^17^ and MIni CArbon DAting System (MICADAS)^18,19^ developed at ETH Zurich. However, the hybrid nature of the MICADAS system offers an alternative solution to the complex process of graphitising small samples. Organic samples containing <100 μg C can be placed into an elemental analyser (EA) directly coupled to the gas ion source of the MICADAS via the gas interface system (GIS)^15,18,20–24^. The automated system reduces both sample preparation time and the risk of contamination through handling, and has been successfully utilised in environmental and climatic applications^23,25–28^. In our preliminary study^29^ we demonstrated that the gas ion source of the AixMICADAS^30^ is suitable for dating bone collagen CO_2_ samples of <100 μg C back to 35,000 BP (uncalibrated radiocarbon years before AD 1950).

However, as sample size is reduced the effect of contamination during pretreatment and measurement increases greatly. Sample pretreatment involves the extraction and purification of carbon endogenous to the original bone. Any contamination remaining in the sample at the time of dating can lead to erroneous results. The effects become increasingly catastrophic with the increasing age of the sample due to the minute concentrations of residual ^14^C. For example, in a bone extract *ca*. 40,000 BP, 1% modern carbon contamination would skew the resulting ^14^C age by over 7,000 years.

It is standard practice to extract the proteinaceous portion of bone for ^14^C measurement, generally referred to as ‘collagen’^31^. Although collagen forms around 22% weight of modern bone, degradation following death and burial makes collagen extraction increasingly challenging with advancing age^32^. Whilst the minimum threshold for reliable ^14^C dating is generally considered to be 1%^32^, it is common for the collagen portion of Palaeolithic bone to constitute <10% weight. The lower the level of collagen preservation, the more bone must be pretreated to obtain sufficient material to assess the quality of the extract (i.e. isotopic and elemental analysis) and for ^14^C dating. Therefore, 300–1000 mg material is commonly sampled for dating Palaeolithic bones.

The majority of ^14^C labs follow collagen extraction protocols based on Longin^33^. This involves demineralisation of either powdered bone or bone chunks using hydrochloric acid (HCl) followed by gelatinisation of the collagen in weakly acidic water and freeze-drying of the final extract. Different labs vary in the strength of reagents used, the duration of treatments and the inclusion of further decontamination steps. Many studies have been published comparing the collagen yields and isotopic values of the various extraction protocols published in the literature^34–38^ as variations in pretreatment conditions can lead to differences in the quantity and quality of the final extracts. The addition of an ultrafiltration step, first proposed by Brown, *et al*.^39^ has in particular improved the accuracy of ^14^C dating of Palaeolithic bones^40^; gelatinised samples are filtered to concentrate large (>30 kDa) molecules to produce a ‘cleaner’ collagen extract. The technique is not unanimously agreed upon due to the risk of contamination from the humectant-coated filter^41^, the effectiveness of the application^37^ and the loss of collagen during filtration^34^. However, stringent cleaning steps have been established^42–44^ and in many cases the re-dating of ancient bones with ultrafiltration methods has produced much older dates than previous measurements from non-ultrafiltered extracts^40,45,46^. The collagen pretreatment protocol routinely applied to Palaeolithic bone at the Max Planck Institute for Evolutionary Anthropology (MPI-EVA, Leipzig, Germany) is based on a modified Longin plus ultrafiltration protocol^36^ and has a strong track record of obtaining high yields of high quality collagen from *ca*. 500 mg samples of Palaeolithic bone^47^.

The aim of this study was to determine a suitable method to pretreat <100 mg bone material and further investigate if the gas ion source of the AixMICADAS^29,30^ at CEREGE (Centre de Recherche et d’Enseignement de Geosciences de l’Environnement, Aix-en-Provence, France) is suitable for measuring small archaeological bone samples with sufficient accuracy and precision. We investigated the effect of sample size reduction at both the pretreatment and gas measurement stages. Tests were performed on a set of eight archaeological bones ranging from 1% to >10% collagen preservation known to date from >50,000–1,400 BP. Each bone was pretreated multiple times from starting weights of 37–100 mg bone material. Each collagen extract was split and dated multiple times with the gas ion source of the AixMICADAS to test replicability. The gas dates were compared with graphite dates from collagen extracted from >500 mg material of the same bones. We further compared gas dates of *ca*. 30 μg C and *ca*. 90 μg C to explore the effect of sample size on the blank level of the EA-GIS-AMS system. The results demonstrate our ability to obtain accurate and moderately precise radiocarbon dates from <100 μg C extracted from 37–100 mg bone material back to 40,000 BP. The methods described will be used to extract and ^14^C date collagen from precious archaeological bone artefacts with minimal sample destruction.

## Results

### Bone pretreatment

Prior to this study, 500 to 700 mg of each bone had been pretreated using our standard collagen extraction protocol^36^. The extracts were analysed by EA-IRMS at the MPI-EVA to assess their suitability for dating (C%, N%, C:N, δ^13^C, δ^15^N) and were measured at the Klaus-Tschira-AMS lab in Mannheim, Germany (lab code: MAMS). The same collagen extracts from R-EVA 1489, R-EVA 123 and R-EVA 124 were also dated at the AixMICADAS facility to cross-check the ages^29^. The results were used as a reference for the preparation of small (<100 mg) aliquots of bone.

**Figure 1:**
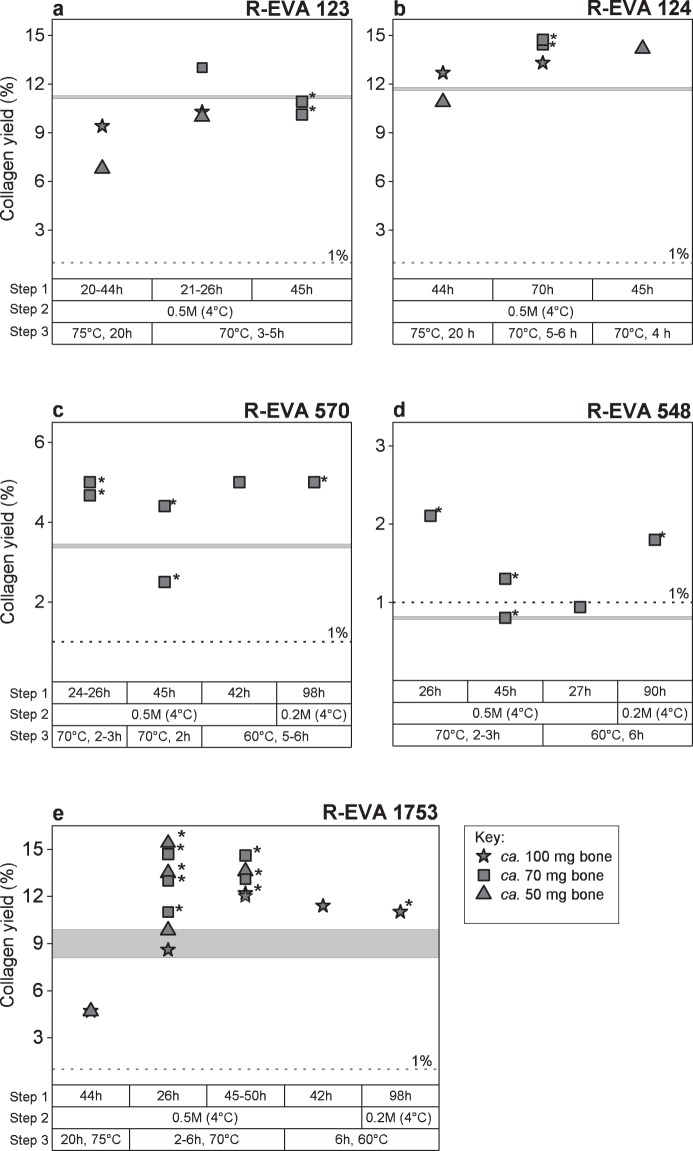
Graphs showing the collagen yields from small aliquots of bone according to variations in pretreatment conditions: (**a**) R-EVA 123, (**b**) R-EVA 124 (**c**) R-EVA 570, (**d**) R-EVA 548 and (**e**) R-EVA 1753. Step 1: duration of the demineralisation stage. Step 2: strength of HCl during demineralisation. Step 3: duration and temperature of the gelatinisation stage (HCl pH3). In (**a**–**d**) the horizontal grey line shows the collagen yield from a large aliquot (>500 mg material) of the same bone. A higher number of data points are present for R-EVA 1753 (**e**) as an aliquot of this bone was extracted alongside each batch of samples. The horizontal grey band in e shows the range in collagen yield of repeated large extractions from the background bone. The dashed lines at 1% show the guideline minimum requirement for reliable ^14^C dating. Asterisks mark extracts which were dated using the gas ion source (see Fig. 3).

Modifications to our standard pretreatment protocol were carried out for five bones (Fig. 1): three relatively ‘well-preserved’ (>10% collagen preservation) archaeological bones (Fig. 1a,b,e) and two ‘poorly-preserved’ bones (<5% collagen preservation) (Fig. 1c,d). Once we had determined the optimum pretreatment protocol for <100 mg material, we applied this to three more archaeological samples: R-EVA 1489, R-EVA 1905 and R-EVA 1860 (two extracts per bone) (pretreatment information shown in Supplementary Dataset S1).

The standard practice in our lab is to extract large bone aliquots (*ca*. 500 mg material) as a solid piece. Although this method requires a large time investment (demineralisation can take up to four weeks with the HCl 0.5 M changed twice per week), we observe much higher collagen yields using this technique compared to powdered extracts of equal starting weight. Small aliquots (<100 mg) of the test bones were initially pretreated as both fine powder and as solid chunks. For solid pieces of bone, in most cases the collagen yield from small extracts (<100 mg) equalled or exceeded the collagen yields of large extracts (500–700 mg material) and no difference was observed between aliquots of 50 mg bone compared to 70 mg or 100 mg bone material (Fig. 1). In contrast, the powdered aliquots of well-preserved bones generally yielded around half the amount of collagen compared to solid pieces, in line with our observation for large starting weights of bone. Powdered aliquots from the poorly preserved bones either yielded nothing or small amounts (<1 mg) of crumbly yellow material. Due to the poor results from the pretreatment of powdered samples, our protocol for small amounts of bone is based on the extraction of solid pieces as per our standard protocol for larger aliquots. The pretreatment information for powdered extracts is included in the supplementary information.

We initially applied our standard collagen extraction protocol to <100 mg bone material of the well-preserved bones. Three steps of the pretreatment protocol were then modified to see what effect this had on the collagen yield and quality of extracts from small bone aliquots (Fig. 1): step (1) the duration of the demineralisation stage; step (2) the strength of HCl during the demineralisation stage; step (3) the temperature and duration of the gelatinisation stage. Bone collagen yields along with elemental (C%, N% and C:N) and stable isotopic data (δ^13^C and δ^15^N) were used to evaluate the extracts from the different methods. In addition, Fourier Transform Infrared Spectroscopy (FTIR) was used to double check the preservation of the extracted collagen, and to detect the presence of possible carbon contaminants^31,48,49^. Detailed pretreatment information for all extracts can be seen in Supplementary Dataset S1.

For the poorly preserved bones (Fig. 1c: R-EVA 570 and Fig. 1d: R-EVA 548) the pretreatment was softened in order to minimise collagen loss during the extraction. The weaker HCl (0.2 M) (step 2) and lower gelatinisation temperature (60 °C) (step 3) required a greater time investment and did not necessarily increase the yield of collagen compared to using stronger acid (HCl 0.5 M) during demineralisation and higher temperatures (70 °C) during gelatinisation. For the poorly preserved samples, demineralisation in HCl 0.5 M generally occurred after one day (4 °C). As Schoeninger, *et al*.^50^ observed that one disadvantage of extracting collagen from solid chunks was the tendency for incomplete demineralisation, several extracts were demineralised in HCl 0.5 M for two days. This resulted in lower collagen yields for the poorly preserved bones and in the case of R-EVA 548, the yield of these extracts was so low that the extracts were affected by C contamination to a large extent.

During the gelatinisation stage (step 3), the collagen yield was higher from aliquots which were removed from the heater block as soon as solubilisation had occurred compared to those left on the heater block for 20 h as per our standard protocol for >500 mg. For all bone samples >30,000 BP, solubilisation occurred in <6 h (Fig. 1), whereas R-EVA 1489 and R-EVA 1905 required up to 27 h for full solubilisation (Supplementary Dataset S1).

**Figure 2:**
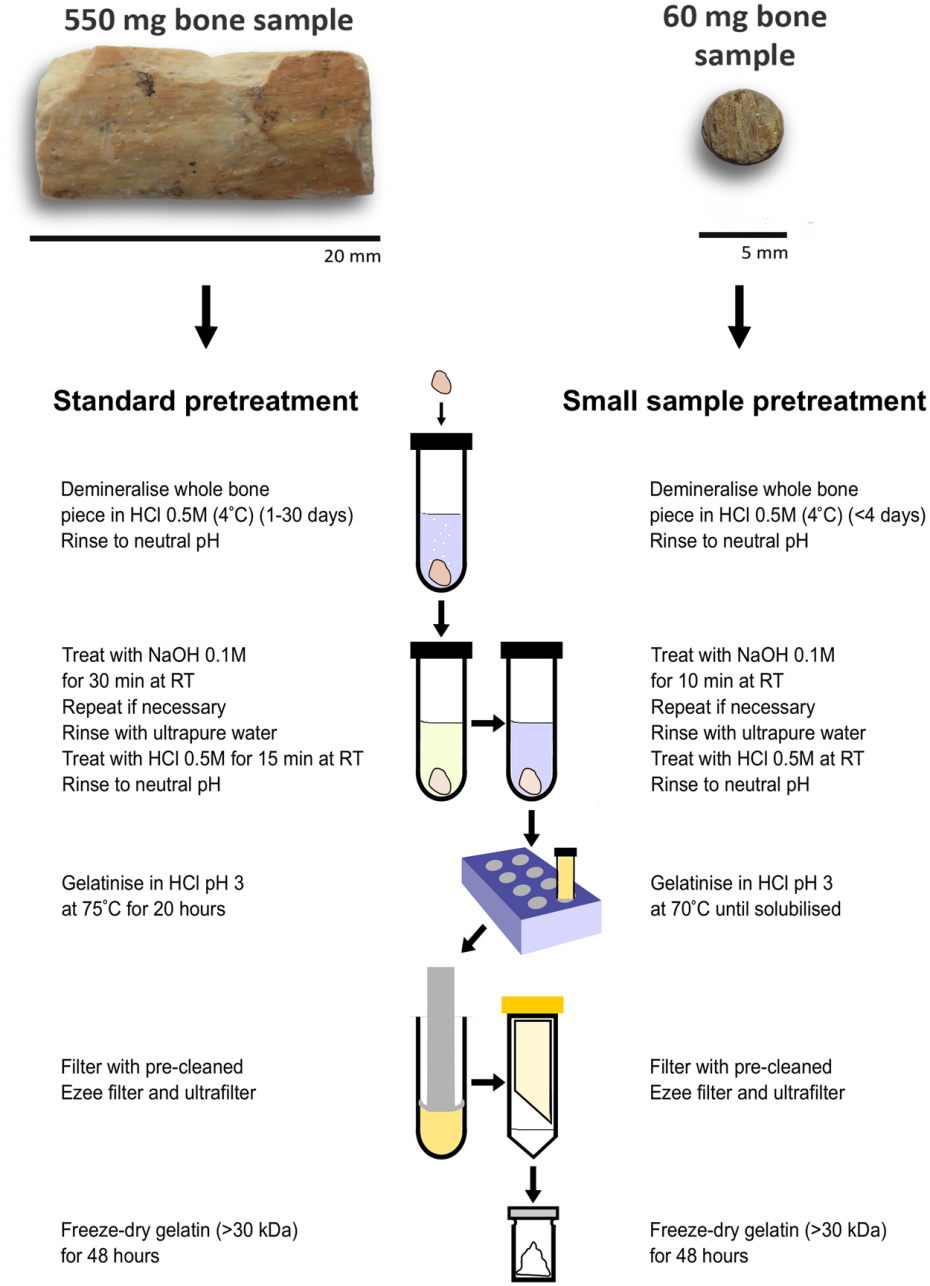
Summary of bone pretreatment protocols used at the MPI-EVA for large (left) and small (right) bone samples.

Of the extracts dated, two (R-EVA 548.13 and R-EVA 548.14) fell close to or under the minimum threshold (1%) for reliable ^14^C dating (Supplementary Dataset S1). There were small variations in elemental values between pretreatments of the same bone but all values (Supplementary Dataset S1) fell within the accepted ranges of ‘well-preserved’ collagen^32^. The stable isotopic values were in keeping with the palaeodietary expectations for each animal and were consistent between extracts. Analysis with FTIR was performed for all collagen extracts; each extract dated had a spectra characteristic of well-preserved collagen when compared to library spectra (see Supplementary Fig. S3). Considering the collagen yields and ^14^C measurements, the optimum pretreatment protocol for small aliquots of bone (<100 mg) is shown in Fig. 2.

### ^14^C dating

**Figure 3:**
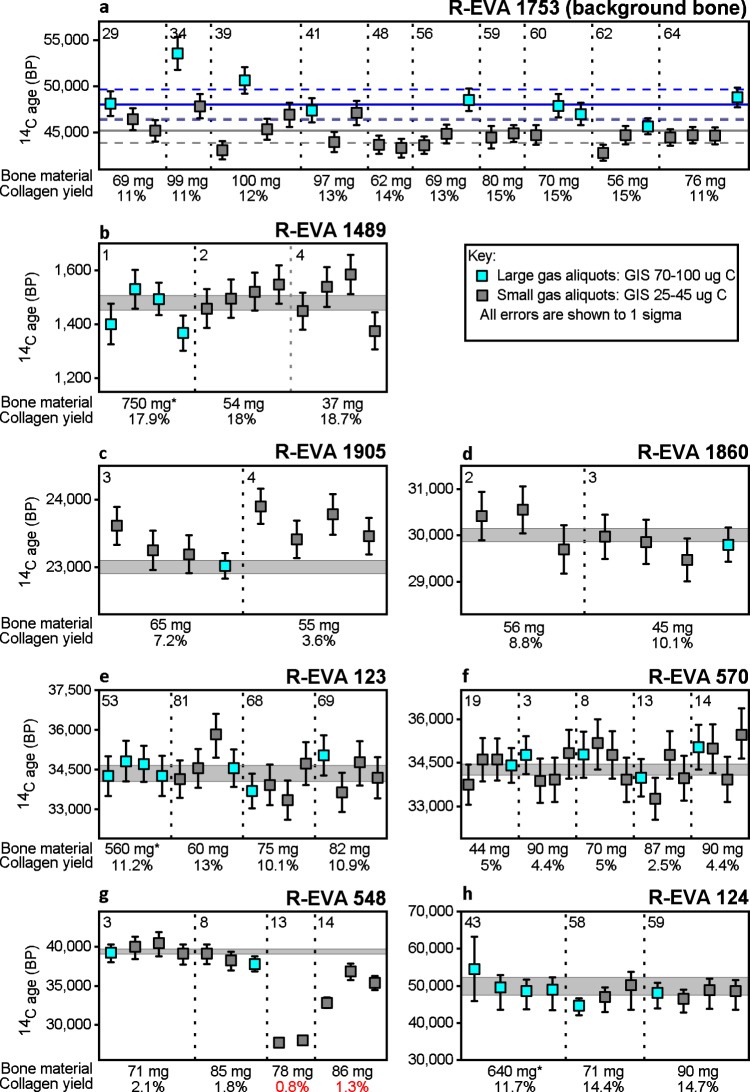
^14^C gas measurements of small (25–40 μg C) and large (70–100 μg C) aliquots of collagen extracted from eight bones (**a**–**h**) spanning the ^14^C time range. Each data point shows the ^14^C age (BP) and 1σ error (years) of a single EA-GIS-AMS measurement. a) Shows the uncorrected measurements of background bone R-EVA 1753 (>50,000 BP). An aliquot of this bone was prepared alongside every batch of samples from sampling to measurement to monitor contamination introduced during sample preparation. These measurements were used in the age calculation of the other archaeological samples (**b**–**h**), according to session, size (small or large) and type (solid bone extract). The arithmetic mean and associated SD of system blank (IAEA-C1/phthalic anhydrite) measurements are shown as a solid horizontal blue line and dashed blue lines respectively for large 80–100 μg C measurements and as a solid horizontal grey line and dashed grey line for small 25–40 μg C measurements. For all gas measurements in graphs b-h: the absolute error of the blank has been set to 0.001 and an external error of 3.5‰ has been added to all measurements based on the long term standard deviation of standards. Dates **>**15,000 BP have been rounded to the nearest 10 years. Asymmetrical errors are shown where F14C ≤ 1σ*10. Grey shaded bands show the 1σ range of graphite dates measured from large extracts of the same bone. In a-h, the vertical dotted lines separate different collagen extracts of the same bone with the bone starting weight and collagen yield shown below. The number in the top left of each section is the preparation number of the bone, corresponding to Supplementary Dataset S1. Asterisks mark collagen extracts dated with the gas ion source reported in Fewlass, *et al*.^29^.

For each of the bones, several collagen extracts (bone weight ranging from 37–100 mg, marked with asterisks in Fig. 1) were dated using the EA-GIS-AixMICADAS (Fig. 3). Each collagen extract was split and measured multiple times. Between two and four replicates were measured containing *ca*. 30–40 μg C, run for the duration of one titanium (Ti) target (*ca*. 12 minutes) and for each bone >20,000 BP, a single aliquot containing *ca*. 80–90 μg C was measured over the duration of three targets to increase precision (see Supplementary Dataset S2). The gas ages obtained were compared to one or more graphite dates measured from collagen extracted from 500–700 mg bone material (Supplementary Dataset S2). Discussed here are measurements made from collagen extracted from solid pieces of bone. Details of measurements made from powdered aliquots (lower collagen yields) are included in the supplementary information.

Figure 3 shows the ages obtained for each bone. The accuracy of the dates generated by the gas ion source is clearly seen in comparison with the graphite dates. Of the 74 new measurements made with the EA-GIS-AMS system shown in Fig. 3b–h, 69 measurements agree within the 95% confidence limit (2σ) of the corresponding graphite dates and 57 agree within 1σ. There are five measurements outside 2σ: four are measurements of the two collagen extracts (R-EVA 548.13; R-EVA 548.14) which fell at or below the minimum threshold of preservation suitable for ^14^C dating (Fig. 3g), and the last (R-EVA 1905.4.1; Aix-12023.2.1) is slightly older than the other replicates of the same extract (Fig. 3c).

Chi-squared tests (χ^2^)^51^ were performed using the R_Combine feature in OxCal 4.2^52^ using the F^14^C and associated error for gas replicates of each collagen extract individually and for all replicates per bone. The replicate measurements are statistically indistinguishable for R-EVA 1489, R-EVA 1905, R-EVA 1860, R-EVA 123, R-EVA 570 and R-EVA 124 (output of all statistical tests are included in Supplementary Dataset S2), demonstrating the reproducibility of the measurements and consistency between different pretreatment batches across the range of the ^14^C timescale. In addition, all of the measurements of R-EVA 1489, R-EVA 123 and R-EVA 124 from this study agree with the EA-GIS-AMS measurements made in 2016 reported in Fewlass, *et al*.^29^ (Supplementary Dataset S2).

The exception is the roughly 40,000 year old bone R-EVA 548, which at *ca*. 1% collagen preservation represents the limits of C^14^ dating. The gas dates obtained from the two low yield extracts (R-EVA 548.13 and R-EVA 548.14) were much younger than the other extracts of this bone (Fig. 3g), showing they had been affected by contamination from modern carbon. Due to the low yield, under normal circumstances R-EVA 548.13 would not have been passed for dating following pretreatment. Excluding these two extracts, the replicates from R-EVA 548.3 and R-EVA 548.8 are consistent with the graphite date for this bone.

For background bone R-EVA 1753 (>50,000 BP), the dates from the collagen extracts (Supplementary Dataset S3) were on par with the blank standards (IAEA-C1/phthalic anhydride) of equal size (Supplementary Dataset S4). As expected, the blank level in the EA-GIS system was affected by the reduction in sample size from 90 μg C to 30 μg C (Fig. 3a). The ages of the seven <50,000 BP samples were corrected with background collagen measurements of the same size (*ca*. 30 μg C or *ca*. 90 μg C) and type (solid/powder) measured during the same session.

## Discussion

Using a slightly modified version of our standard pretreatment protocol the collagen yield from <100 mg bone material was of equally high quality as extracts from ‘large’ (>500 mg) bone samples. Decreasing sample size from *ca*. 100 mg to <50 mg bone material also had no detrimental effect on collagen yield. The agreement in age between multiple collagen extracts from different starting weights of bone (Fig. 3) indicates firstly that we obtain reproducible results with the pretreatment protocol and secondly, that the reduction in material during pretreatment did not detrimentally affect the results of ^14^C dating. In particular, the results indicate that the cleaning steps used for the ultrafilters are sufficient as any C remaining in the filters after cleaning would have a more pronounced effect on reduced sample sizes.

The main alteration to our standard protocol involved reduction in the duration of the gelatinisation stage, with samples removed from the heater block as soon as they had gelatinised (see Fig. 2). Different gelatinisation conditions have been well documented to affect the final extract quality and yield^38,39,53,54^. The higher collagen yields from these extracts supports observations that gelatinised collagen is degraded by prolonged exposure to higher temperatures and acidity^39,53^.

R-EVA 548 represents a very challenging prospect for collagen extraction and radiocarbon dating due to the exceptionally low levels of preservation (<1% weight collagen) and old age (*ca*. 39,400 BP), even working with larger sample sizes. The harshest demineralisation (HCl 0.5 M, 2 days, 4 °C) applied to small aliquots of this bone (R-EVA 548.13; R-EVA 548.14) resulted in very low yields of ≤1 mg collagen, likely due to the solubilisation of collagen during the longer demineralisation stage. The resultant underestimated dates clearly show that these aliquots were massively affected by modern carbon contamination. Prior to dating, the consideration of the quality of the extract is crucial in order to obtain reliable dates. Given the low yield of collagen (≤1%) following pretreatment, under normal circumstances these extracts would not been dated or would have been treated with caution. This bone demonstrates the difficulty of pretreatment of poorly preserved bones at the limit of the ^14^C method.

At such small sample sizes, the consideration of the background correction is crucial. The gas measurements of R-EVA 1489, R-EVA 1905, R-EVA 1860, R-EVA 123, R-EVA 570, R-EVA 548 and R-EVA 124 were all corrected with gas measurements of background bone collagen (R-EVA 1753) of equal size (*ca*. 30 µg C or *ca*. 90 µg C) prepared alongside every batch of samples and measured during the same measurement session to account for any C added during sample preparation and measurement. Figure 3a shows the ages obtained for the background bone containing *ca*. 25–40 μg C (small) and *ca*. 80–100 μg C (large). The large measurements (mean F^14^C = 0.0024, SD = 0.0006, n = 9, equivalent to 48,600 BP) are on par with the system blank (either IAEA-C1 or phthalic anhydride) measurements of equal size (mean F^14^C = 0.0026, SD = 0.0006, n = 7, equivalent to 48,000 BP) (Supplementary Datasets S3 and S4), indicating that no carbon contamination was introduced during sample preparation. An increased sensitivity to modern ^14^C is to be expected at lower levels of carbon and it is clear that the smaller background collagen measurements are generally younger. The 25–40 μg C background collagen samples (mean F^14^C = 0.0039, SD = 0.0007, n = 22, equivalent to 44,530 BP) are likewise equal to the system blank measurements of equal size (mean F^14^C = 0.0036, SD = 0.0006, n = 5, equivalent to 45,180 BP) (Supplementary Datasets S3 and S4). These values are lower than previously published values for blank IAEA-C1 samples measured at CEREGE reported in Bard, *et al*.^30^ (F^14^C = 0.02 for sample sizes around 30 µg C and F^14^C = 0.005 for samples of 80–100 µg C) and to phthalic anhydride blanks measured at ETH Zurich reported in McIntyre, *et al*.^24^ (mean F^14^C = 0.0046 ± 0.0012, n = 6, size range 84–100 μg C). The results indicate the lower limit of ^14^C detection with the gas ion source to be around F^14^C = 0.004. As demonstrated by R-EVA 124, beyond this limit the minute levels of ^14^C can be measured but the uncertainty of the background correction dominates accuracy and precision.

The system blank of the EA-GIS-AMS is affected by the carbon content of the silver cups, cross-talk of the zeolite trap and the cleanliness of the ion source at the time of the measurement^24^. The mass (M_c_) and F^14^C (F^14^C_c_) of the constant contamination of the EA + GIS system was deduced by least square regression of modern carbonate and blanks (IAEA-C1) with sample weights ranging between 3 and 100 µg C to be M_c_ = 0.55 ± 0.05 ug C and F^14^C_c_ = 0.12 ± 0.03^55^. The silver cups (5 × 3 mm from Elementar; cleaned at 800 °C, 2 h) had a consistent carbon contribution of 0.049 ± 0.02 µg C. The zeolite trap was heated (450 °C) and the system was flushed with helium between samples to minimize cross-contamination. However, small amounts of C may reside in the zeolite trap after flushing which has been demonstrated to have a large influence on samples <20 µg carbon^23,55^. With this in mind, even our ‘small’ samples were kept >20 μg carbon. To further alleviate problems of cross-talk, samples were run in order of increasing activity (oldest to youngest) according to the standard practice^55^. Background corrections of samples were applied according to sample size and an external error was added during the age calculation of all samples based on the long term standard deviation of standards and blanks (error 2 described in Fewlass, *et al*.^29^).

In a real life situation, if a small bone sample yielded a high amount of collagen (for example, the mammoth bone R-EVA 123 or the Medieval human bone R-EVA 1489 included in this study), dating with graphite targets would be preferentially undertaken as the precision achieved is much higher and measurements can be made routinely. However, the results of this study demonstrate that the gas ion source can produce an accurate radiocarbon date at low precision from as little as 30 µg C. The precision of the date can be improved when larger sample sizes (up to 100 µg C) are available for measurement over several targets (as demonstrated in Fig. 3). In order to assess variability in handling and blank contribution, in this study we compared multiple measurements of *ca*. 30 µg C with larger aliquots containing *ca*. 90 µg C. When taking the weighted mean and error of the three small aliquots the precision achieved is higher compared to the single large measurement of a roughly equal amount of carbon. However, as the likelihood of contamination being introduced via handling, the EA-GIS or the silver cup is increased for the smaller sample sizes, the preferred method for measuring larger samples would be to measure several targets from a single syringe, rather than splitting a sample into smaller aliquots. Although the measurement of gas samples requires more supervision than graphite targets, the direct coupling of the EA with the GIS significantly reduces sample preparation time by cutting out the graphitisation step which poses a large risk of contamination at such small sample sizes. Therefore in situations where sample size is limited the gas ion source offers an attractive solution for archaeological, as well as environmental, applications.

Even working with the assumption of 1% collagen preservation, in theory sufficient collagen could be extracted from less than 10 mg bone material to obtain a ^14^C date using the EA-GIS-AMS. However in order to assess the quality of the extract prior to dating and obtain high-resolution stable isotopic data for palaeodietary reconstruction, collagen should also be analysed with an EA-IRMS. At 1%, around 40 mg bone material would supply enough collagen for dating and isotopic analysis. For any sample >1% preservation, excess collagen would be available for further analyses and/or multiple aliquots could be measured with the gas ion source to achieve better counting statistics and thus increase precision. Bearing this in mind, when dating highly precious bone it would be useful to assess the preservation of the artefact prior to sampling or have an understanding of collagen preservation at the archaeological site (for example if other fauna has been sampled for isotopic or ^14^C dating purposes). Bones of high patrimonial value could be sampled strategically – i.e. for older samples expected to have less than 10% collagen preservation 40 mg bone material could be sampled, whereas for well-preserved Holocene bone much smaller samples could be taken. The case of R-EVA 548 demonstrates that for very old samples (>35,000 BP) with very poor levels of preservation (1–2%), yields falling below 1 mg collagen can be subject to severe contamination issues.

The results presented here provide further confirmation that ^14^C measurements using the gas ion source of the MICADAS are stable, reproducible and accurate, reaching a level of precision suitable for dating archaeological samples particularly for Palaeolithic samples back to 40,000 BP. In this respect this technique will be highly useful for directly dating precious archaeological bone where limited material is available.

## Methods

### Sample selection

Eight bones were selected to span the ^14^C timescale (back to 50,000 BP) at a range of preservation typical for archaeological bones. Collagen extracts from bones R-EVA 124, R-EVA 123 and R-EVA 1489 were previously dated using both graphite targets and the gas ion source in Fewlass, *et al*.^29^. R-EVA 124 was previously labelled as a bison bone but recent aDNA analysis has identified it as belonging to a woolly rhinoceros^56^. R-EVA 548 and R-EVA 570 are two faunal long bones from Teixoneres, Spain. R-EVA 1860 is a faunal long bone excavated from the site of Ranis, Germany and R-EVA 1905 is a predominantly trabecular fragment of horse bone excavated from Pietraszyn, Poland. R-EVA 1753 is a well-preserved cave bear rib known to date beyond the ^14^C timescale based on repeated measurements. As standard practice, an aliquot of this bone is extracted and dated alongside every batch of samples to monitor contamination introduced during sample preparation and is used in the age correction of the unknown samples. This is the referred to in the text as the ‘background bone’.

### Collagen extraction

For each bone, large aliquots (500–700 mg material) were pretreated using our standard acid-base-acid + gelatinisation + ultrafiltration protocol (see Fig. 2) based on Talamo and Richards^36^ to produce collagen for dating with graphite targets.

In order to optimise our standard protocol for sample sizes <100 mg, small aliquots of each bone were pretreated multiple times to compare collagen yields and sample quality. Firstly, the outer surface of bone was removed using a sandblaster and aliquots were taken using a rotary drill. Fine diamond grit disc drill pieces were used to remove solid pieces of bone. Fine powder was drilled using round tungsten carbide burs (2.3 mm diameter). Aliquots were weighed via a microbalance into cleaned glass tubes. Solid samples were demineralised in HCl at 4 °C with regular visual and mechanical checks and monitoring of CO_2_ effervescence. For powdered samples, HCl was added and samples were monitored at room temperature (RT) until CO_2_ effervescence had stopped. Following demineralisation, samples were rinsed with ultra-pure Milli-Q water to a neutral pH. Samples were treated with NaOH (0.1 M) at RT for 10 min to remove humic acid contamination and re-acidified with HCl (0.5 M). If a considerable colour change was observed, NaOH was changed and left for another 10 min. Samples were then gelatinised in weak HCl (pH 3) on a heater block set to 60 °C, 70 °C or 75 °C. Samples were either left for 20 h (as per our standard pretreatment), or regularly monitored and removed from the heater block when the sample had fully solubilised. The resultant gelatin was filtered to remove large particles >80 µm (Ezee filters, Elkay labs, UK) and ultrafiltered with Sartorius VivaSpin Turbo 15 (30 kDa MWCO) ultrafilters precleaned according to Brock, *et al*.^43^ to separate the high molecular weight fraction (>30kD) for freeze drying (48 h). For details of acid strength, duration of treatment and temperature during pretreatment of samples <100 mg, see Fig. 1 and Supplementary Dataset S1.

### Collagen quality assessment

To assess the quality of the collagen, all extracts were analysed via EA-IRMS to obtain elemental (C%, N%, C:N) and stable isotopic data (δ^13^C and δ^15^N). Collagen (*ca*. 400 μg) was weighed into tin cups using a microbalance and measured on a ThermoFinnigan Flash EA coupled to a Thermo Delta plus XP isotope ratio mass spectrometer (IRMS). Stable carbon isotope ratios were expressed relative to VPDB (Vienna PeeDee Belemnite) and stable nitrogen isotope ratios were measured relative to AIR (atmospheric N_2_), using the delta notation (δ) in parts per thousand (‰). Repeated analysis of both internal and international standards indicates an analytical error of 0.2‰ (1σ) for δ^13^C and δ^15^N. Where sufficient material was available, collagen (*ca*. 300 μg) was homogenized and mixed with ∼40 mg of IR grade KBr powder in an agate mortar and pestle, pressed into a pellet using a manual hydraulic press (Wasserman) and analysed with an Agilent Technologies Cary FTIR Spectrometer with a DTGS detector. Spectra were recorded in transmission mode at 4 cm^−1^ resolution with averaging of 34 scans between 4000 and 400 cm^−1^ using Resolution Pro software (Agilent Technologies). The spectra were evaluated and compared to library spectra of well-preserved collagen and bone to look for evidence of incomplete demineralisation, degraded collagen or the presence of any exogenous material in the extracts.

### AMS graphite measurements

Each bone was pretreated as per our standard protocol from approximately 500 mg material. From theses extracts, approximately 3–5 mg collagen was weighed into pre-cleaned tin cups at the MPI-EVA and sent to the Curt-Engelhorn-Centre for Archaeometry Klaus-Tschira-AMS facility in Mannheim, Germany (lab code: MAMS) for graphite dating. The samples were combusted in an EA and the sample CO_2_ was converted catalytically to graphite. The samples were dated using the MICADAS-AMS^57^. Age and error calculation of unknown samples was performed using BATS software^58^, using background collagen samples and standards measured in the same batch, with an added external error of 1‰ as per their standard practice. Collagen samples measured at CEREGE were weighed into tin cups (*ca*. 2 mg), combusted in a vario MICRO cube EA (Elementar Analysensysteme GmbH, Germany), graphitized using the AGE 3 and dated using the AixMICADAS. Oxalic acid standards and background collagen samples measured in the same session were used to calculate the age of the samples. An external error of 1‰ was also propagated in the error calculation.

### AMS gas ion source measurements

Small aliquots (<100 mg) of the same bones were pretreated to purify the collagen. Three or four aliquots of each collagen extract (containing *ca*. 25–40 μg C and a single aliquot per bone containing *ca*. 80–100 μg C) were measured via a microbalance into pre-cleaned silver cups (800 °C, 2 h). These were placed into the auto-sampler of a vario MICRO cube EA which was directly coupled to the gas ion source of the AixMICADAS via the GIS^20,22^. Following combustion, sample CO_2_ was adsorbed on a zeolite trap and subsequently expanded to the syringe of the GIS where it was mixed with He (5% CO_2_) and introduced to the gas ion source at a flow rate of *ca*. 2 µg C/min. The EA-GIS system was flushed with helium between samples. Pre-cleaned titanium (Ti) gas targets were pre-sputtered for approximately two minutes in the ion source to remove any remaining surface contamination before the sample CO_2_ injection. Around 30–40 µg C was consumed by the AMS over the duration of one Ti target^21,55^. For the large aliquots containing *ca*. 80–90 μg C measurements were performed over multiple targets (which can be changed during measurement). Each step was fully controlled via the gas-interface handling software.

The gas measurements in this study were made over two measurement sessions six months apart, both carried out shortly after the ion source had been cleaned. Each measurement session commenced with two oxalic acid II NIST standards (from a gas canister) to normalize and correct samples for fractionation. Blank (^14^C-free) CO_2_ samples (also from a gas canister) were then measured to purge the system and reach a stable operational level (F^14^C < 0.004) (these measurements were not used in age calculation). In the first session, carbonate reference material (IAEA-C1) were run prior to the collagen samples to check the background level of the instrument and begin the measurement of old samples under optimal conditions. In the second measurement session, phthalic anhydride was run for the same purpose. In order to alleviate problems of memory effect, the GIS system was flushed with helium between samples and samples were measured in order of increasing activity as per standard procedure (for further discussion, see Tuna, *et al*.^55^). Low energy ion currents for the gas analyses were in the range of 10–15 μA. BATS^58^ was used for data reduction. The uncorrected collagen background (R-EVA 1753) measurements of the corresponding type (piece/powder) and equal size were used to correct the archaeological samples measured in the same session (i.e. ‘small’ sample aliquots were corrected only with ‘small’ background collagen samples). For all samples, the long term standard deviation of blanks (F^14^C = 0.001) was used as the absolute blank error and an external error of 3.5‰ was added to take into account the long-term variability of standards (‘error 2’ described in Fewlass, *et al*.^29^).

## Supplementary information

Supplementary text and figures

Dataset S1

Dataset S2

Dataset S3

Dataset S4

## Data Availability

All data generated or analysed during this study are included in this article and the accompanying supplementary information files.

## References

[1] Oakley KP (1963). Dating Skeletal Material. Science.

[2] Berger R, Horney AG, Libby WF (1964). Radiocarbon Dating of Bone and Shell from Their Organic Components. Science.

[3] Wood R (2015). From revolution to convention: the past, present and future of radiocarbon dating. Journal of Archaeological Science.

[4] Pearson A (1998). Microscale AMS 14C Measurement at NOSAMS. Radiocarbon.

[5] Hua Q, Zoppi U, Williams AA, Smith AM (2004). Small-mass AMS radiocarbon analysis at ANTARES. Nuclear Instruments and Methods in Physics Research Section B: Beam Interactions with Materials and Atoms.

[6] Santos GM, Southon JR, Griffin S, Beaupre SR, Druffel ERM (2007). Ultra small-mass AMS 14C sample preparation and analyses at KCCAMS/UCI Facility. Nuclear Instruments and Methods in Physics Research Section B: Beam Interactions with Materials and Atoms.

[7] Smith AM, Petrenko VV, Hua Q, Southon J, Brailsford G (2007). The Effect of N2O, Catalyst, and Means of Water Vapor Removal on the Graphitization of Small CO2 Samples. Radiocarbon.

[8] Genberg J, Stenstrom K, Elfman M, Olsson M (2010). Development of graphitization of μg-sized samples at Lund University. Radiocarbon.

[9] Delqué-Količ E (2013). Advances in Handling Small Radiocarbon Samples at the Laboratoire de Mesure du Carbone 14 in Saclay, France. Radiocarbon.

[10] Liebl J (2013). Carbon background and ionization yield of an AMS system during C-14 measurements of microgram-size graphite samples. Nuclear Instruments and Methods in Physics Research Section B: Beam Interactions with Materials and Atoms.

[11] Walter SRS (2015). Ultra-Small Graphitization Reactors for Ultra-Microscale C-14 Analysis at the National Ocean Sciences Accelerator Mass Spectrometry (NOSAMS) Facility. Radiocarbon.

[12] Freeman E, Skinner LC, Reimer R, Scrivner A, Fallon S (2016). Graphitization of Small Carbonate Samples for Paleoceanographic Research at the Godwin Radiocarbon Laboratory, University of Cambridge. Radiocarbon.

[13] Steier P, Liebl J, Kutschera W, Wild EM, Golser R (2016). Preparation Methods of μg Carbon Samples for 14C Measurements. Radiocarbon.

[14] Ertun T (2005). Progress in AMS target production of sub-milligram samples at the NERC radiocarbon laboratory. Radiocarbon.

[15] Ruff M (2010). Gaseous radiocarbon measurements of small samples. Nuclear Instruments and Methods in Physics Research Section B: Beam Interactions with Materials and Atoms.

[16] Cersoy S (2017). Radiocarbon dating minute amounts of bone (3–60 mg) with ECHoMICADAS. Scientific Reports.

[17] Wacker L, Němec M, Bourquin J (2010). A revolutionary graphitisation system: Fully automated, compact and simple. Nuclear Instruments and Methods in Physics Research Section B: Beam Interactions with Materials and Atoms.

[18] Ruff M (2007). A gas ion source for radiocarbon measurements at 200 kV. Radiocarbon.

[19] Wacker L (2010). MICADAS: Routine and High-Precision Radiocarbon Dating. Radiocarbon.

[20] Ruff M (2010). On-line radiocarbon measurements of small samples using elemental analyzer and MICADAS gas ion source. Radiocarbon.

[21] Fahrni SM, Wacker L, Synal HA, Szidat S (2013). Improving a gas ion source for 14C AMS. Nuclear Instruments and Methods in Physics Research Section B: Beam Interactions with Materials and Atoms.

[22] Wacker L (2013). A versatile gas interface for routine radiocarbon analysis with a gas ion source. Nuclear Instruments and Methods in Physics Research Section B: Beam Interactions with Materials and Atoms.

[23] Wacker L, Lippold J, Molnár M, Schulz H (2013). Towards radiocarbon dating of single foraminifera with a gas ion source. Nuclear Instruments and Methods in Physics Research Section B: Beam Interactions with Materials and Atoms.

[24] McIntyre CP (2016). Online 13C and 14C Gas Measurements by EA-IRMS–AMS at ETH Zürich. Radiocarbon.

[25] Bonvalot L (2016). Estimating contributions from biomass burning and fossil fuel combustion by means of radiocarbon analysis of carbonaceous aerosols: application to the Valley of Chamonix. Atmos. Chem. Phys..

[26] Hoffmann, H. M. *Micro radiocarbon dating of the particulate organic carbon fraction in Alpine glacier ice: method refinement*, *critical evaluation and dating applications*, Universität Heidelberg (2016).

[27] Gottschalk J (2018). Radiocarbon Measurements of Small-Size Foraminiferal Samples with the Mini Carbon Dating System (Micadas) at the University of Bern: Implications for Paleoclimate Reconstructions. Radiocarbon.

[28] Haghipour N (2019). Compound-Specific Radiocarbon Analysis by Elemental Analyzer–Accelerator Mass Spectrometry: Precision and Limitations. Analytical Chemistry.

[29] Fewlass H (2017). Size Matters: Radiocarbon Dates of <200 µg Ancient Collagen Samples with AixMICADAS and Its Gas Ion Source. Radiocarbon.

[30] Bard E (2015). AixMICADAS, the accelerator mass spectrometer dedicated to 14C recently installed in Aix-en-Provence, France. Nuclear Instruments and Methods in Physics Research Section B: Beam Interactions with Materials and Atoms.

[31] DeNiro MJ, Weiner S (1988). Chemical, enzymatic and spectroscopic characterization of “collagen” and other organic fractions from prehistoric bones. Geochimica et Cosmochimica Acta.

[32] van Klinken GJ (1999). Bone Collagen Quality Indicators for Palaeodietary and Radiocarbon Measurements. Journal of Archaeological Science.

[33] Longin R (1971). New Method of Collagen Extraction for radiocarbon Dating. Nature.

[34] Jørkov MLS, Heinemeier J, Lynnerup N (2007). Evaluating bone collagen extraction methods for stable isotope analysis in dietary studies. Journal of Archaeological Science.

[35] Pestle WJ (2010). Chemical, elemental, and isotopic effects of acid concentration and treatment duration on ancient bone collagen: an exploratory study. Journal of Archaeological Science.

[36] Talamo S, Richards M (2011). A comparison of bone pretreatment methods for AMS dating of samples >30,000 BP. Radiocarbon.

[37] Fulop R-H, Heinze S, John S, Rethemeyer J (2013). Ultrafiltration of bone samples is neither the problem nor the solution. Radiocarbon.

[38] Cersoy S, Zazzo A, Lebon M, Rofes J, Zirah S (2016). Collagen Extraction and Stable Isotope Analysis of Small Vertebrate Bones: A Comparative Approach. Radiocarbon.

[39] Brown TA, Nelson DE, Vogel JS, Southon JR (1988). Improved collagen extraction by modified longin method. Radiocarbon.

[40] Higham T (2011). European Middle and Upper Palaeolithic radiocarbon dates are often older than they look: problems with previous dates and some remedies. Antiquity.

[41] Hüls CM, Grootes PM, Nadeau MJ (2009). Ultrafiltration: Boon or Bane?. Radiocarbon.

[42] Bronk Ramsey C, Higham T, Bowles A, Hedges R (2004). Improvements to the pretreatment of bone at Oxford. Radiocarbon.

[43] Brock F, Bronk Ramsey C, Higham T (2007). Quality assurance of ultrafiltered bone dating. Radiocarbon.

[44] Brock F, Higham T, Ditchfield P, Bronk Ramsey C (2010). Current pretreatment methods for ams radiocarbon dating at the oxford radiocarbon accelerator unit (orau). Radiocarbon.

[45] Higham TFG, Jacobi RM, Ramsey CB (2006). AMS radiocarbon dating of ancient bone using ultrafiltration. Radiocarbon.

[46] Wood RE (2013). Radiocarbon dating casts doubt on the late chronology of the Middle to Upper Palaeolithic transition in southern Iberia. Proceedings of the National Academy of Sciences.

[47] Hublin JJ (2012). Radiocarbon dates from the Grotte du Renne and Saint-Cesaire support a Neandertal origin for the Chatelperronian. Proceedings of the National Academy of Sciences.

[48] Yizhaq M (2005). Quality Controlled Radiocarbon Dating of Bones and Charcoal from the Early Pre-Pottery Neolithic B (PPNB) of Motza (Israel). Radiocarbon.

[49] D’Elia M (2007). Evaluation of possible contamination sources in the 14C analysis of bone samples by FTIR spectroscopy. Radiocarbon.

[50] Schoeninger MJ, Moore KM, Murray ML, Kingston JD (1989). Detection of bone preservation in archaeological and fossil samples. Applied Geochemistry.

[51] Ward GK, Wilson SR (1978). Procedures for Comparing and Combining Radiocarbon Age Determinations: A Critique. Archaeometry.

[52] Bronk Ramsey C (2009). Bayesian Analysis of Radiocarbon Dates. Radiocarbon.

[53] Semal P, Orban R (1995). Collagen Extraction from Recent and Fossil Bones: Quantitative and Qualitative Aspects. Journal of Archaeological Science.

[54] Brock F, Geoghegan V, Thomas B, Jurkschat K, Higham TFG (2013). Analysis of Bone “Collagen” Extraction Products for Radiocarbon Dating. Radiocarbon.

[55] Tuna T, Fagault Y, Bonvalot L, Capano M, Bard E (2018). Development of small CO2 gas measurements with AixMICADAS. Nuclear Instruments and Methods in Physics Research Section B: Beam Interactions with Materials and Atoms.

[56] Korlević P, Talamo S, Meyer M (2018). A combined method for DNA analysis and radiocarbon dating from a single sample. Scientific Reports.

[57] Kromer B, Lindauer S, Synal HA, Wacker L (2013). MAMS - A new AMS facility at the Curt-Engelhorn-Centre for Achaeometry, Mannheim, Germany. Nuclear Instruments and Methods in Physics Research Section B: Beam Interactions with Materials and Atoms.

[58] Wacker L, Christl M, Synal HA (2010). Bats: A new tool for AMS data reduction. Nuclear Instruments and Methods in Physics Research Section B: Beam Interactions with Materials and Atoms.

