# Supplementary material for: Pretreatment and gaseous radiocarbon dating of 40–100 mg archaeological bone
Source: Sci Rep. 2019 Mar 29;9:5342. doi: 10.1038/s41598-019-41557-8 (PMC6440986; doi:10.1038/s41598-019-41557-8)
Supplement: Supplementary file 1 — Supplementary text and figures [file 41598_2019_41557_MOESM1_ESM.pdf]

Pretreatment and gaseous radiocarbon dating of 40-100 mg archaeological bone

Fewlass, H.[1], Tuna, T.[2], Fagault, Y.[2], Hublin, J-J. [1], Kromer, B.[1,3], Bard, E.[2], Talamo, S.[1]

Supplementary information:

- Table S1 – Pretreatment information for all collagen extracts in the study
- Table S2 – 14C dates for all samples in the study dated with graphite targets and the gas ion source
- Table S3 – EA-GIS-AMS data from background bone R-EVA 1753
- Table S4 – EA-GIS-AMS data from system blanks
- Figure S1 – Gas dates from background bone R-EVA 1753 according to the amount of C in the EA-GIS
- Figure S2 – Gas measurements of R-EVA 123 and R-EVA 124 from both solid and powdered extracts
- Figure S3 - Example FTIR spectra of collagen extracted in the study

Pretreatment and ^{14}C dating of powdered bone samples

Bone aliquots were extracted in two forms: fine powder and solid pieces (as per our standard protocol for >500mg bone). We attempted to extract collagen from finely powdered bone to increase our sampling options for precious bones (i.e. a key hole drilling technique). However, the collagen yield of powdered bone was much lower than solid pieces for all samples in the study (Supplementary Fig. S2; Supplementary Table S1). Where collagen was recovered often the extracted material appeared poorly preserved with a crumbly texture and was often dark grey or yellow in colour. Where enough material was available for analysis, these extracts were still identified as collagen when analysed with FTIR (Supplementary Fig. S3), although several extracts from the poorly preserved bones showed evidence of incomplete demineralisation. Anecdotally, the striking difference between the two forms was observed at the demineralisation stage; for the older, poorly preserved bones much of the powdered material was lost as soon as HCl was added to the tube. Although the powdered method has the benefit of being faster, increased solubilisation of collagen during demineralisation in powdered bones compared to solid bone sherds was also observed by Schoeninger, et al. [1] and Collins and Galley [2]. As the length of demineralisation is based on visual inspection, a suitable duration is much easier to judge for solid pieces (transparency, softness, buoyancy, CO_2 effervescence).

As a consequence of the low yield of collagen for the poorly preserved bones (R-EVA 570 and R-EVA 548) no powdered extracts from these bones were dated. Despite the lower collagen yield, sufficient collagen was available for gas dating from powdered aliquots of the well-preserved bones, R-EVA 123, R-EVA 124 and R-EVA 1753. The age of the background collagen extracts were slightly younger than their solid counterparts (Supplementary Fig. S1) but it is unknown whether this reflected the limited number of measurements made, the lower collagen yield from these pretreatments and/or the small size of the aliquots measured in the EA-GIS-AMS (*ca*. 25 µg C).The ages of the <50,000 BP samples were corrected with background collagen measurements of the same size (*ca*. 30 µg C or *ca.* 90 µg C) and type (solid/powder) measured during the same session. The exception to this are the large (*ca.* 90 µg C) powder samples from R-EVA 123 and R-EVA 124 (Aix-12002.7.1; 12002.8.1; 12003.8.5; 12003.9.5) which are marked with an asterisk in Supplementary Figure S2. No background measurement of corresponding size/type was made so these were corrected with small (*ca.* 30 µg C) powder backgrounds meaning they are slightly over-corrected. Even with this over-correction, the age of Aix-12002.8.1 is younger than other measurements for this bone. We do not have an explanation for this measurement.

Despite this, there is no difference between the gas measurements obtained from powdered versus solid extracts for R-EVA 123 or R-EVA 124, which all agree within X^2 despite the over-corrected samples (Supplementary Fig. S2; Supplementary Table S2). Further, the gas dates from the powdered extracts of R-EVA 123 and R-EVA 124 all agree with the graphite dates within 2σ. However, due to the reduced collagen yield we will

continue our standard practice of extracting collagen from solid chunks of bone (also documented in Tuross [3]).

References

1 Schoeninger, M. J., Moore, K. M., Murray, M. L. & Kingston, J. D. Detection of bone preservation in archaeological and fossil samples. *Applied Geochemistry* **4**, 281-292, doi:10.1016/0883-2927(89)90030-9 (1989).

2 Collins, M. J. & Galley, P. Towards an optimal method of archaeological collagen extraction: The influence of pH and grinding. *Ancient Biomolecules* **2**, 209 (1998).

3 Tuross, N. Comparative Decalcification Methods, Radiocarbon Dates, and Stable Isotopes of the Viri Bones. *Radiocarbon* **54**, 837-844 (2012).

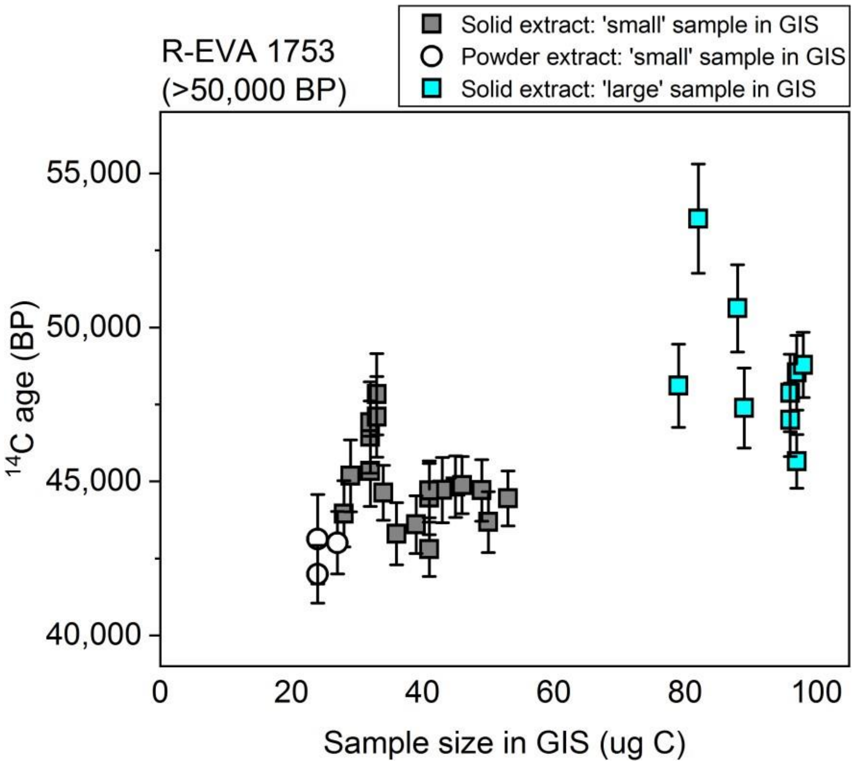

Figure S1 Gas measurements of collagen from background bone R-EVA 1753 according to the amount of carbon in the EA-GIS system. Error bars are shown to 1σ.

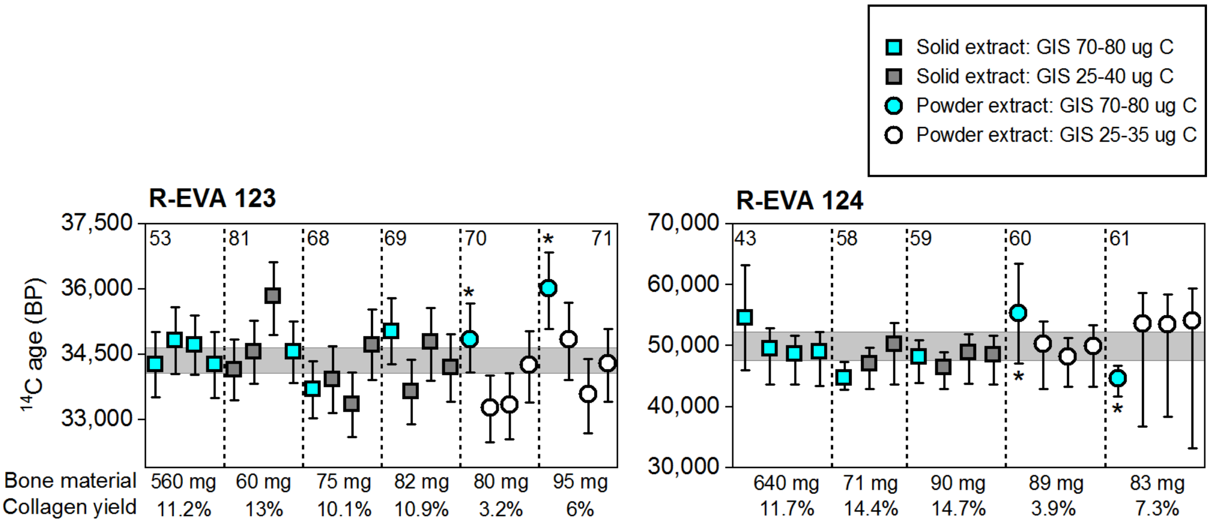

Figure S2 Gas measurements of collagen from R-EVA 123 and R-EVA 124. Figure amended from Figure 3 in the main text to include gas measurements of collagen extracted from powdered bone.

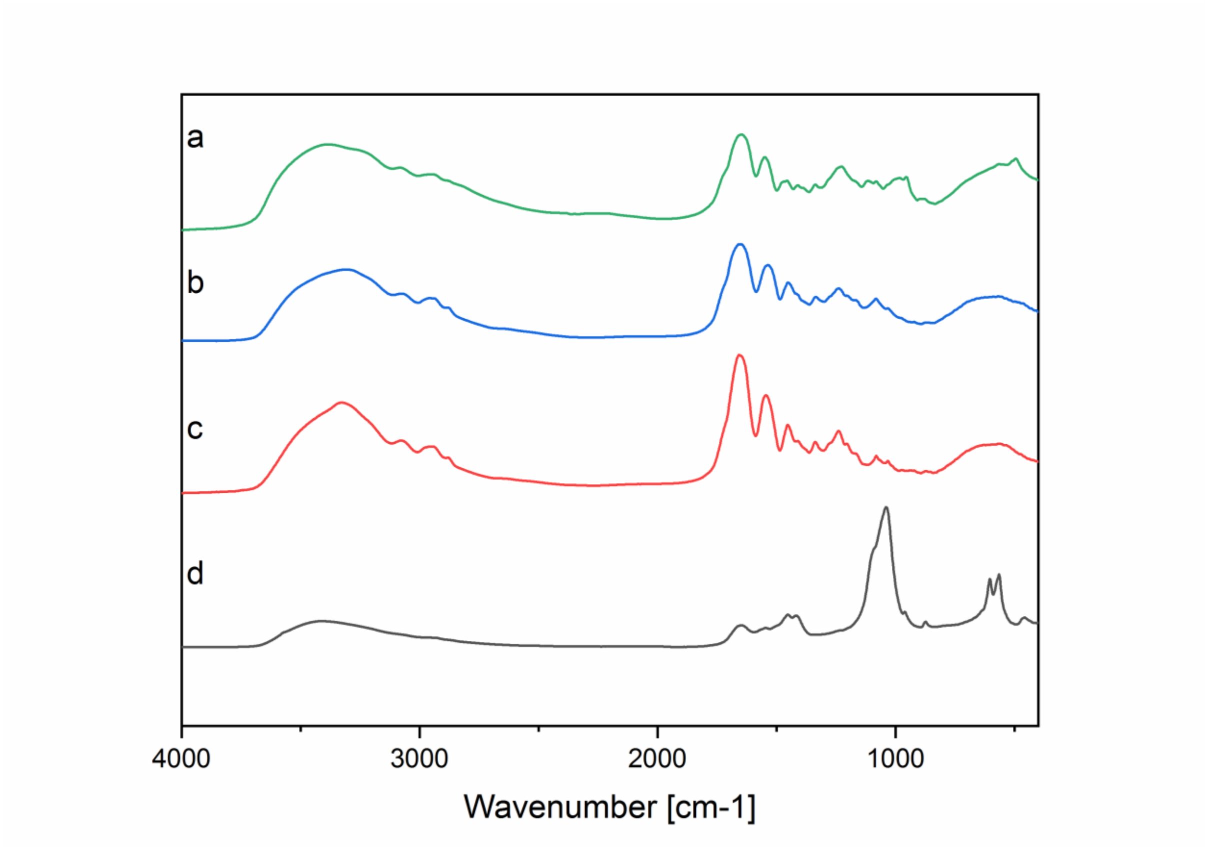

Figure S3 FTIR spectra of collagen extracted from a) R-EVA 570.15 (powder) and b) R-EVA 1489.2 (solid) in comparison to characteristic FTIR spectra of c) well-preserved collagen and d) bone.
